# Multidisciplinary Contributions to Legume Crop History: Proceed with Caution

**DOI:** 10.3389/fpls.2016.01876

**Published:** 2016-12-20

**Authors:** Frank M. Dugan

**Affiliations:** USDA-ARS Plant Introduction, Washington State UniversityPullman, WA, USA

**Keywords:** archeobotany, crop history, paleolinguistics, pulses, legumes

Accurate understanding of crop biogeography facilitates comprehension of agronomic potential, genetic diversity, and crop–pathogen evolution. Classic perspectives are exemplified by Vavilov (1987), a posthumous compilation, and Harlan (1971). Origin and geographic spread of a given crop provide clues as to environmental interactions, including relative adaption to pests, pathogens, and abiotic factors (Dark and Gent, 2001; Dugan, 2015). Great antiquity of pulse crops (pea, chickpea, lentil, bitter vetch, faba bean) is documented archeobotanically for the Fertile Crescent and several adjacent areas, including much of Europe in succeeding times (e.g., Abbo et al., 2003, 2006; Zohary et al., 2012; Mikić et al., 2014).

These crops are also indicated in ancient texts of the Fertile Crescent and adjacent areas, e.g., Akkadian, Old Babylonian (Semitic languages), Hittite (Indo-European, IE), and Sumerian (reviewed in Dugan, 2015). Linguistic indications of pulse crops in ancient Greek, Latin, Old Slavic, etc., and more recent languages have been summarized (Mikić, 2012). However, there remain areas in which data are scarce or in which there is conspicuous dissent from consensus. This is especially true for the Bronze Age Steppes, now seen as critically important in dissemination of language, culture, and peoples (Haak et al., 2015), and possibly the geographic location for the ancestral Proto-Indo-Europeans (Anthony, 2007).

Archeobotanical data for the region remain suboptimal due to the infrequency of applying modern techniques for recovery and dating of plant remains (Mallory, 2014), but repeated instances of cereal chaff imprints or chaff itself on pottery (~4500 to 3500 BC) imply cropping of cereals (Motuzaite-Matuzeviciute, 2012). Presence of pulse crops is not yet conclusively documented, and they may have been absent, although they are present in the archeobotanical record for regions bordering the Steppes (Dugan, 2015). Archeobotanical evidence for pulses appears lacking in the Steppes when archeobotanical sites with recovery of pulses are mapped (Mikić, 2012, 2015a, 2016; Mikić et al., 2014). One possible explanation for archeological recovery of remains of cereals in the Steppes, but not of pulses, is that Bronze Age Steppes were semiarid, as indicated by paleoclimatic studies (Alekseeva et al., 2007; Khomutova et al., 2007; Mitusov et al., 2009). Estimates of mean annual precipitation imply climate may have been unsuitable for consistent cultivation of legumes, but typically with sufficient moisture for cereals (Dugan, 2015).

We have no literature in Proto-Indo-European (PIE, a reconstructed language), analogous to the agricultural writings of ancient civilizations. But perhaps analyses of PIE can promote understanding of Bronze Age agriculture with regard to pulses. Initial inspection of agronomic literature gives reason to believe so. Mikić (2016), indicates a putatively PIE root ^*^erə*g*^*w*^[*h*] – (“a kernel of a leguminous plant”) as originating in the Steppes just northeast of the Black Sea. Mikić (2015a,b) attributes to PIE additional words for legumes, including pea and lentil, as do Mikić (2009, 2011, 2012) and Mikić et al. (2008). The notion that PIE contains words for legumes is now embedded in agronomic literature, including this journal (Mikić, 2015c). Cited in justification (e.g., in Mikić, 2009, 2012) are Vasmer (1953), the slightly more recent Pokorny (1959, 1969) and a website, The Tower of Babel^1^. The website references “Fraenkel 358” for PIE ^*^lent- (lentil), and also “Pokorny's dictionary.” Fraenkel (1962) contains “lent” on page 358, but not as PIE and not lentil. I could not locate the putative PIE terms from Mikić (2009, 2012) in Vasmer (1953), although Vasmer provides Slavic roots similar to these terms. Also, “Pokorny (1959) is badly out of date; moreover it errors extravagantly on the side of inclusion, listing every word …that might conceivably reflect a PIE lexeme” (Ringe, 2006). It therefore becomes necessary to inspect literature from contemporary linguists specifically addressing PIE, and in doing so a different result is obtained. We find that words for cereals were abundant in PIE (Mallory and Adams, 1997, 2006), but the case is more ambiguous for pulses.

At present, the consensus seems to be that words for pulses appear in later regional terms (Indo-European, but not PIE) or represent borrowings (“loan words”) from non-Indo-European languages. Mallory and Adams (1997) note with regard to pea, “our inability to reconstruct in a solid way any PIE word for it.” These authors state for vetch (*Vicia sativa*), bitter vetch (*Vicia ervila*), and grass pea (*Lathyrus sativus*), “there is no evidence for IE antiquity [i.e., PIE] for any of these crops [and] the lentil (*Lens culinaris*) is also unretrievable from the IE lexicon.” Mallory and Adams (2006) state with regard to pea and chickpea, “their designations are found only regionally …(Latin, Greek), which raises the possibility that they may derive from a non-IE substratum.” Somewhat more ambiguous is a possible PIE reconstruction for bean, ^*^bhabheh_*a*_-, although Mallory and Adams (2006) indicate this word as among “regional terms” eventually yielding Greek, Latin, and Albanian equivalents. That names of pulses in Greek, Latin, and some other IE languages originate from a Pre-Greek, *non*-Indo-European substratum has long been the opinion (Sturtevant, 1910; Mann, 1943; Hester, 1968), and this consensus has held (e.g., Adrados, 2005; Kroonen, 2012; Darden, 2013). This does not mean that Proto-Indo-Europeans were utterly unfamiliar with pulses, but consensus among those just cited is that words specifically designating pulses are *not* found in reconstructed PIE.

Origins of the Proto-Indo-Europeans and PIE have long been sought in Anatolia (Renfrew, 1990; Bouckaert et al., 2012) or, in a competing hypothesis, the Steppes of Ukraine and Russia (Anthony, 2007). The Steppes and Anatolian hypotheses are relevant to historical biogeography of agricultural crops. Absence, or near absence, of PIE words denoting pulses, and the lack, or near lack, of archeobotanical records for pulses in the Bronze Age Steppes, would correlate with paleoclimatic data indicating that the Steppes of the Bronze Age were generally too arid for successful cultivation of pulses. Both the Steppes and Anatolia provided adequate conditions for grazing livestock and for cereals (Hald, 2010; Motuzaite-Matuzeviciute, 2012), all of which are repeatedly manifest in PIE. Anatolia, however, also provided conditions adequate for pulses, and pulses are substantially present in the archeobotanical record there (e.g., Sadori et al., 2006). If the origins of PIE are sought in Anatolia, the consistent lack of PIE words for pulses, as noted by Mallory, Adams and their colleagues, requires explanation. If Mikić and his colleagues are correct, no such explanation is necessary under the Anatolian hypothesis.

An accurate understanding of pulses in Proto-Indo-European and Indo-European agriculture and language will have positive consequences for our understanding of archeology, linguistics, and crop biogeography. A compilation of PIE agricultural terms is forthcoming in proceedings from a conference in Leipzig (J. Mallory and G. Kroonen, editors; J. Mallory, personal communication).

## Author contributions

The author confirms being the sole contributor of this work and approved it for publication.

### Conflict of interest statement

The author declares that the research was conducted in the absence of any commercial or financial relationships that could be construed as a potential conflict of interest.

## Footnotes

^1^http://starling.rinet.ru/.
